# A Comprehensive Model of Factors Associated With Capability to “Live Well” for Family Caregivers of People Living With Mild-to-Moderate Dementia: Findings From the IDEAL Study

**DOI:** 10.1097/WAD.0000000000000285

**Published:** 2018-12-05

**Authors:** Linda Clare, Yu-Tzu Wu, Catherine Quinn, Ian R. Jones, Christina R. Victor, Sharon M. Nelis, Anthony Martyr, Rachael Litherland, James A. Pickett, John V. Hindle, Roy W. Jones, Martin Knapp, Michael D. Kopelman, Robin G. Morris, Jennifer M. Rusted, Jeanette M. Thom, Ruth A. Lamont, Catherine Henderson, Isla Rippon, Alexandra Hillman, Fiona E. Matthews

**Affiliations:** * Centre for Research in Ageing and Cognitive Health (REACH), School of Psychology; ‡ Wellcome Centre for Cultures and Environments of Health, University of Exeter; † PenCLAHRC, University of Exeter Medical School; ¶ Innovations in Dementia, Exeter; § Wales Institute for Social and Economic Research, Data, and Methods, Cardiff University, Cardiff; ∥ College of Health and Life Sciences, Brunel University London; # Alzheimer’s Society; §§ Personal Social Services Research Unit, London School of Economics and Political Science; Departments of ∥∥ Psychological Medicine; ¶¶ Psychology, King’s College London, Institute of Psychiatry, Psychology, and Neuroscience, London; ** Department of Care for the Elderly, Betsi Cadwaladr University Health Board, Llandudno; †† School of Psychology, Bangor University, Bangor; ‡‡ RICE (The Research Institute for the Care of Older People), Bath; ## School of Psychology, University of Sussex, Brighton; ††† Institute for Health and Society, Newcastle University, Newcastle upon Tyne, UK; *** School of Medical Sciences, University of New South Wales, Sydney, Australia

**Keywords:** quality of life, satisfaction with life, well-being, health, stress

## Abstract

**Introduction:**

Understanding key influences on outcomes for caregivers of people with dementia is hampered by inconsistent conceptualization and measurement of outcomes and limited evidence about the relative impact of different variables. We aimed to address these issues.

**Methods:**

We analyzed data from 1283 caregivers of community-dwelling individuals with mild-to-moderate dementia in the Improving the experience of Dementia and Enhancing Active Life cohort study. We generated a “living well” latent factor from measures of quality of life, satisfaction with life, and well-being. We used structural equation modelling to derive latent variables for 7 domains reflecting caregivers’ perceptions of their personal resources and experiences, and to examine the associations with caregivers’ perceptions of their capability to “live well.”

**Results:**

The domain of psychological characteristics and psychological health was most strongly related to living well [2.53; 95% confidence interval (CI), 2.08-2.97], followed by physical fitness and physical health (1.48; 95% CI, 1.04-1.91) and experiencing caregiving (1.34; 95% CI, 0.99-1.70). Social capitals, assets and resources (0.68; 95% CI, 0.35-1.00) and relationship with the person with dementia (−0.22; 95% CI, −0.41 to −0.03) had smaller, significant associations. Social location (0.28; 95% CI, −0.33 to 0.89) and managing everyday life with dementia (0.06; 95% CI, −0.15 to 0.28) were not significantly associated with living well.

**Discussion:**

These findings demonstrate the importance of supporting caregivers’ psychological and physical health and their ability to develop and maintain positive coping strategies, as well as enabling them to maintain vital social capitals, assets and resources.

## Abstract 2

Supplemental Digital Content is available in the text.

Family members and friends (here together termed “caregivers”) provide vital unpaid care and support for people with dementia living in the community. In 2015 the global cost of informal care for people with dementia was estimated at $330.8 billion, representing 40.4% of the total costs attributed to dementia.^1^^p60^ In the United Kingdom, caregivers provide 1.34 billion hours of unpaid care to people with dementia each year, equating to a cost of £11.6 billion or 44% of the total cost of dementia care.^2^

People with dementia have needs for care which are different to, and greater than, those of other groups with long-term health problems or disabilities, and these evolve and increase over time as the disease progresses.^3^ Compared with both the general population and caregivers supporting people with other illnesses or disabilities, caregivers of people with dementia experience poorer quality of life (QoL),^4^,^5^ satisfaction with life^6^ and well-being.^7^ These are all indices of capability to “live well” while providing care. “Living well” means experiencing the best possible level of well-being, reflected in a subjective sense of “comfort, function, and contentment with life.”^8^^p32^

Caring for a person with dementia can bring many challenges to the ability to live well,^9^ including loss and grief, isolation and loneliness, depression, anxiety and exhaustion, and the demands of providing care may need to be balanced against the caregiver’s other responsibilities or own health problems. The potential stresses of providing family care and their impact on the caregiver have been captured in the influential stress process model of caregiving^10^ and the concept of caregiver burden.^11^ Equally, however, it has been noted that burden is not a strong predictor of QoL,^12^ and other factors have a role to play. Well-being among those caring for a person with dementia is more closely associated with the characteristics of the caregiver and the caregiving situation than with the illness characteristics of the person with dementia or the objective level of burden.^13^ Caregivers’ own resources, including social support^14^ and personal strengths such as personality traits,^15^ an optimistic outlook and feelings of competence^16^ and use of problem-focused rather than emotion-based coping strategies^17^ underpin maintenance of mental health and are associated with better well-being. Furthermore, it is increasingly recognized that caregiving itself can lead to positive experiences, such as accomplishment and enjoyment of the role, feelings of mutuality, increased family cohesion, and personal growth and purpose in life.^18^–^20^

A comprehensive understanding of these positive and negative influences could inform knowledge about how to provide effective support for caregivers of people with dementia. However, evidence about potential influences on indices of capability to live well is relatively limited. A recent synthesis included 41 studies and examined the associations between 47 variables, grouped into 10 themes, and caregiver QoL.^21^ This demonstrated that caregiver QoL is affected by multiple factors. The authors point out that heterogeneity among studies precluded calculation of standardized effect sizes and direct comparisons, and the number of statistically significant associations reported in studies may have been inflated due to reliance on correlational analyses without correction for multiple comparisons. Thus, it was not possible to identify how variables may be interrelated or determine which variables or groups of variables demonstrate the strongest associations.

Furthermore, in caregiving literature the terms QoL, satisfaction with life and well-being are frequently conflated or used interchangeably at both conceptual and measurement levels.^12^,^22^–^26^ Although numerous studies have attempted to identify associations between individual variables and one of these key measures, given that these 3 concepts are correlated and show similar associations with key variables,^27^,^28^ combining them into a single factor might demonstrate stronger and more consistent associations across a wider range of variables.

In this study we aimed to address this inconsistency in concepts and measures by combining standardized measures of QoL, satisfaction with life, and subjective well-being into a single “living well” factor and comprehensively modelling influences on capability to “live well” for caregivers. Using data from the Improving the experience of Dementia and Enhancing Active Life (IDEAL) cohort study,^29^,^30^ we grouped 48 potentially associated variables into 7 domains, used multivariate modelling to derive latent variables for these domains, and utilized structural equation modelling to examine how these domains were associated with each other and with “living well” for caregivers of people with mild-to-moderate dementia.

## METHODS

### Design

IDEAL is a longitudinal cohort study of people living with dementia and their caregivers throughout Great Britain (England, Scotland, and Wales). Trained interviewers visit participants’ homes and conduct face-to-face interviews with the person with dementia while participating caregivers self-complete the questionnaire booklets. As part of the consent process it is made clear that participants with dementia and caregivers may choose not to answer any questions they prefer not to address. An involvement group of people living with dementia and caregivers, the Action on Living Well: Asking You (ALWAYs) group, advises on the design and conduct of the study and contributes to interpreting the results. This analysis is based on cross-sectional data from the first wave of data collection and uses version 2.0 of the data set. IDEAL was approved by the Wales Research Ethics Committee 5 (reference 13/WA/0405), and the Ethics Committee of the School of Psychology, Bangor University (reference 2014—11684). IDEAL is registered with the UK Clinical Research Network (UKCRN), number 16593.

### Participants

The participants in this study are the caregivers of people living with dementia recruited into the IDEAL cohort. People with dementia living in the community were recruited through memory clinics and other specialist services in 29 National Health Service sites throughout England, Scotland, and Wales, and through the online Join Dementia Research portal, between July 2014 and August 2016. Inclusion criteria were a clinical diagnosis of dementia and a Mini-Mental State Examination^31^ score of ≥15, and ability to provide informed consent. Exclusion criteria were other terminal illness and any significant risk to interviewers conducting home visits. In total 1547 people with dementia agreed to participate. Approximately 67% lived in urban and 33% in rural areas.^32^ When a person with dementia joined the study, where available a caregiver was invited to take part as well. For the purposes of the study “caregiver” was defined as the main family member or friend providing unpaid care and support to the person with dementia. There were no other specific inclusion or exclusion criteria for caregivers. In total 1283 caregivers agreed to participate; of these 1045 (81.5%) lived with the participating person with dementia.

### Measures of Capability to “Live Well”

The outcomes explored in this analysis were caregivers’ subjective perceptions of QoL, satisfaction with life and well-being, combined into a single latent factor of “living well.” QoL was assessed with the World Health Organization QoL-BREF (WHOQOL-BREF).^33^ The 26 items cover 4 domains physical health, psychological, social relationships, and environment, plus 2 general questions, and are rated on a 1 to 5 scale. The mean score for items within each domain is used to calculate the domain score. The domain score is then multiplied by 4 to give a score of 100. For the purposes of the present analysis, to derive a single QoL score, the 4 domains and 2 general questions were included in a factor analysis model and a predicted factor score was derived for those with complete information. Satisfaction with life was assessed with the Satisfaction with Life Scale (SwLS).^34^ The 5 items are rated on a 1 to 7 scale and responses are added to give a total score of 35. Higher scores indicate greater life satisfaction. Well-being was assessed with the World Health Organization-Five Well-Being Index (WHO-5).^35^ The 5 items are rated on a 0 to 5 scale and responses are added to give a total score of 25, which is multiplied by 4 to give a score of 100. Higher scores indicate greater well-being.

### Measures of Potential Predictor Variables

The 7 life domains addressed in the IDEAL study caregiver interview covered 48 possible predictor variables which were considered for inclusion in the structural equation model (SEM). The 7 domains were as follows:

- Social capitals, assets and resources—this reflected social resources, social networks, and participation in social and cultural activities.
- Social location—this reflected socioeconomic status and perceived social standing.
- Psychological characteristics and psychological health—this reflected psychological traits and dispositions, including personality characteristics, optimism, self-esteem and self-efficacy, as well as loneliness and depression.
- Physical fitness and physical health—this reflected physical activity, lifestyle, and health conditions.
- Managing everyday life with dementia—this included perceived severity of and distress at symptoms, and involvement of the person with dementia in decision making.
- Relationship with the person with dementia—this included assessments of current and past relationship quality.
- Experiencing caregiving—this reflected both positive and challenging aspects of caregiving, such as competence, coping, stress, and social restriction.

Supplementary Table 1 (Supplemental Digital Content 1, http://links.lww.com/WAD/A211) summarizes the variables considered under each domain, and how these were measured.

### Statistical Methods

Within each of the 7 life domains, univariable multivariate modelling was used to select variables for inclusion in the SEM. The relationship of each variable with the 3 outcome measures was first examined individually, and statistical significance and clinical relevance were considered. Statistical significance was investigated with the Wald test. The effect size for a given variable was considered to be meaningful if unstandardized regression coefficients were >1.5 for SwLS^36^ and >5 for WHO-5;^37^ there was no applicable cut-off for the WHOQOL-BREF factor score. Variables from each domain that were influential in multivariate modelling were included in the latent factor for that domain within the SEM.

The SEM estimated a latent factor for each domain and structural associations between different latent factors and “living well” in caregivers, adjusting for age, sex, caregiver relationship with the person with dementia, and dementia subtype. The percentage of missing data ranged from 7% to 20% across all domains. Multiple imputation was conducted to account for missing data including all variables in the modelling. Ten imputed data sets were generated and combined using Rubin’s rule. The model was parameterized to reflect positive associations indicating enhanced “living well” outcomes. A coefficient estimate was assumed to be significant if its 95% confidence interval (CI) did not include 0 (see the Supplementary Information, Supplemental Digital Content 1, http://links.lww.com/WAD/A211, for further details).

## RESULTS

### Participant Characteristics

Characteristics of the caregivers are summarized in Tables 1 and 2. Table 2 also provides mean scores on the QoL-AD, SwLS, and WHO-5. Men tended to report higher scores than women on all 3 of these measures. Spouses and partners had lower well-being and QoL than other family members or friends, but similar levels of satisfaction with life. Satisfaction with life appeared to increase with age but this pattern was not seen in QoL or well-being. Caregivers of people with Parkinsonian dementias had lower scores on all 3 measures than caregivers of people from other diagnostic groups.

**TABLE 1:**
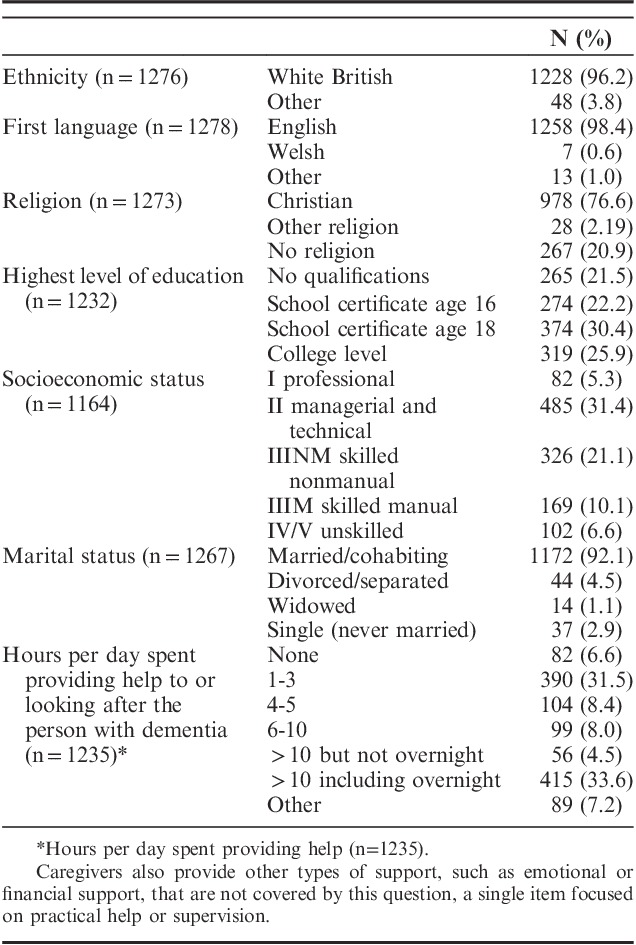
Characteristics of the Caregivers (n=1283)

**TABLE 2:**
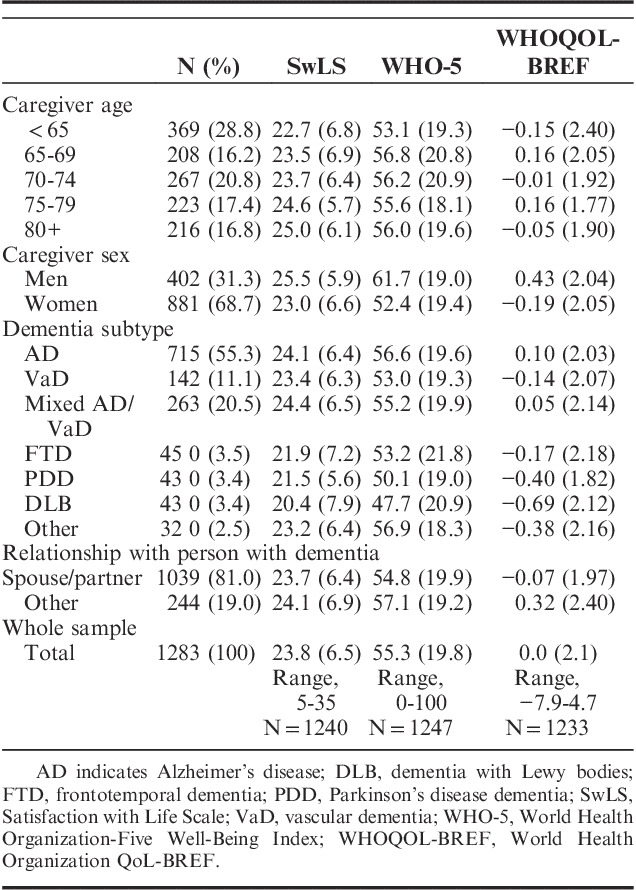
Means and SDs of Scores on “Living Well” Measures for the Whole Sample and by Caregiver Age, Sex, Relationship to Person with Dementia and Dementia Subtype

### Variables Included in the Analysis

The variables retained for inclusion in each domain through univariable multivariate modelling are summarized in Table 3. Full details of the stages of modelling are provided in Supplementary Table 2 (Supplemental Digital Content 1, http://links.lww.com/WAD/A211).

**TABLE 3:**
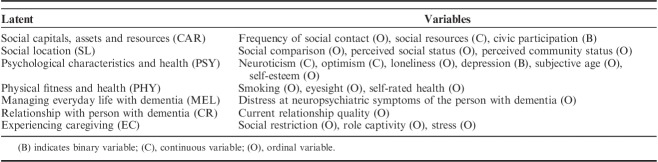
Variables Included in the Latent Factors for Each Domain in the Structural Equation Model

### Relationships Among the Latent Variables

**FIGURE 1:**
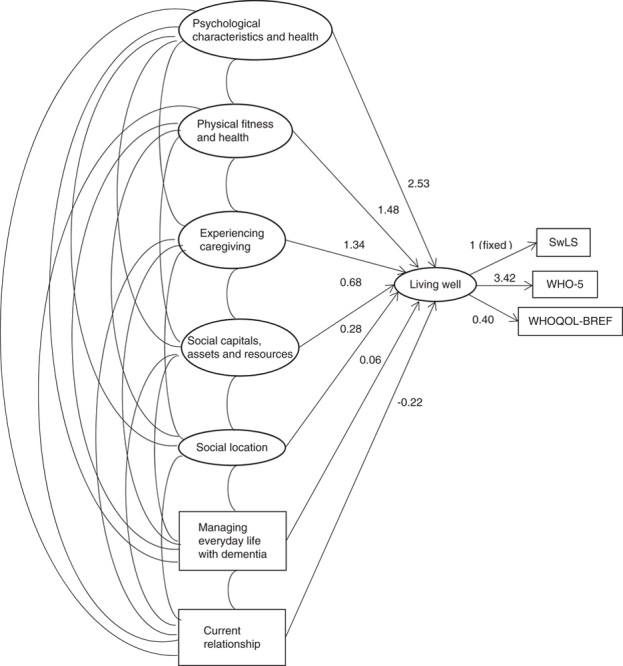
Associations between the 7 domains and caregivers’ QoL, well-being, and satisfaction with life (n=1283; adjusted for age, sex, relationship with person with dementia, and dementia subtype). SwLS indicates Satisfaction with Life Scale; QOL, quality of life; WHO-5, World Health Organization-Five Well-Being Index; WHOQOL-BREF, World Health Organization QoL-BREF.

In the final model, following multiple imputation analysis and with adjustment, psychological characteristics and psychological health was most strongly related to “living well” (2.53; 95% CI, 2.08-2.97), followed by physical fitness and physical health (1.48; 95% CI, 1.04-1.91) and experiencing caregiving (1.34; 95% CI, 0.99-1.70). Social capitals, assets and resources (0.68; 95% CI, 0.35-1.00), and relationship (−0.22; 95% CI, −0.41 to −0.03) had smaller but still significant associations. Social location (0.28; 95% CI, −0.33 to 0.89) and managing everyday life with dementia (0.06; 95% CI, −0.15 to 0.28) were not significantly associated with “living well”. A visual representation of the model is presented in Figure 1, and further detail is provided in Supplementary Table 3 (Supplemental Digital Content 1, http://links.lww.com/WAD/A211).

## DISCUSSION

We have presented a comprehensive model of factors associated with “living well” for a large sample of 1283 caregivers of people with mild-to-moderate dementia, drawn from socially and environmentally diverse areas of Great Britain. This analysis uniquely combined 48 individual predictor variables to derive 7 latent factors reflecting key domains of the caregiving experience, and 3 outcome variables to derive a single “living well” factor, in order to create a model demonstrating the significant associations with “living well” and the relative strength of these associations. The model indicates that caregivers’ psychological characteristics and psychological health are most strongly associated with subjective perceptions of “living well,” while their physical fitness and physical health, and their experience of caregiving, also demonstrate important associations. Social capitals, assets and resources play a more limited but still important role. Social class and perceived social status are not associated with whether or not the caregiver is “living well.” Current relationship with the person with dementia, again, plays a limited though still important role, while dementia-related problems reflected in perceptions of neuropsychiatric symptoms are not associated with “living well.” These findings provide a more integrated understanding of influences on outcomes for caregivers than hitherto available.

Previous research demonstrates that good mental and physical health are consistently associated with caregiver QoL.^21^ These factors are recognized in the stress process model,^10^ where depression, anxiety, and poor physical health are seen as resulting from a combination of objective and subjective stressors and strains. Several studies indicate an association between depression and poorer outcomes for caregivers.^14^,^38^–^40^ Although there has been a considerable focus on depression in previous research, the impact of psychological characteristics has received much less attention. Our modelling incorporated a range of psychological characteristics alongside depression, and the final model included the personality trait of neuroticism, optimism, self-esteem, and the subjective sense of loneliness. Recent work has indicated the relevance of personality traits,^15^ while hope has been identified as related to better QoL,^40^ and optimism has been associated with greater life satisfaction.^16^ Our study builds on this by grouping several psychological factors together and including these alongside other nonpsychological predictors. This provides a stronger foundation for emphasizing the importance of psychological characteristics and psychological health among carers of people with dementia.

Alongside these personal characteristics, we focused on the social capitals, assets and resources that were available to the caregivers in our sample. The final model included frequency of social contact, availability of resources within the caregiver’s social network that could be called upon to address particular needs, and civic participation. Caregivers’ perceptions of social support have been identified previously as important for QoL, satisfaction with life and well-being.^12^,^41^,^42^ A recent review found no clear association with QoL,^21^ but this was based on data from only 2 studies. Our more comprehensive examination of the role of social capitals, assets and resources, in the form of social support and civic participation, reinforces the finding that social support plays an important role in enabling carers to live well.

As regards the caregivers’ experience of caregiving, our model included stress, social restriction, and role captivity. Caregivers’ perceptions of the experience of caregiving are likely to be underpinned by both general psychological characteristics and health and the availability of support and resources. Previous reviews have indicated that stress is related to poorer QoL.^21^,^40^ The caregiver’s perception of the quality of the relationship with the person with dementia was also important in our model. One study^43^ found that closeness in the relationship was associated with better caregiver well-being, but also noted that subsequent decline in closeness over time has a detrimental effect.

Two of 7 domains covered in our modelling did not show significant associations with living well. One of these, managing everyday life with dementia, reflected perceived severity of and distress at neuropsychiatric symptoms. Farina et al^21^ found that the relationship between presence of behavioral and psychological symptoms in the person with dementia and caregiver QoL was unclear, with only about half of the included studies finding an association. The IDEAL cohort included participants who, at baseline, had mild-to-moderate dementia and were living in the community; hence, levels of neuropsychiatric symptoms may have been relatively low, precluding the possibility of finding an association between perceptions of these and outcomes for caregivers, should such an association exist. The other, social location, considered demographic characteristics of the caregiver and our modelling included an objective assessment of socioeconomic status as well as the caregiver’s own perceptions of status in relation to others, and in the community and wider society. The final model included only the caregiver’s perceptions and found no association with “living well”. Similarly, Farina et al^21^ found no strong evidence with regard to associations between demographic characteristics of the caregiver and caregiver QoL.

There are a number of limitations to this study that must be acknowledged. Our data are based largely on self-report, and while this is valuable in capturing caregivers’ perceptions of their own situation and experience, inclusion of objective measures might strengthen the analysis. The analysis is based on cross-sectional data and causal direction cannot be inferred. Selection of variables was necessary in developing the model, and while the variables remaining were those with clear domain-specific relationships, some small effects may have been omitted in the final modelling stage. The participating caregivers were providing care and support to people with mild-to-moderate dementia living in the community, and associations may be different for caregivers of people who have more advanced dementia or who are receiving institutional care. The analysis included all participating caregivers and adjusted for the relationship to the person with dementia. However, the majority of caregivers were spouses or partners, and their experiences may differ from those of adult children or other family members or friends providing care. We were unable to consider cultural and ethnic differences as the sample consisted almost entirely of white British individuals. Caregivers from black and minority ethnic groups may be less likely to access health services and related support^17^ and hence are important to consider.^44^ Our model incorporating 7 domains of experience was developed through consensus of the research team, and reviewed with our ALWAYs group of experts by experience. We acknowledge that while the majority of measures can be readily allocated to a specific domain, there are some measures where opinions could differ on the most appropriate grouping. Future research might test this or similar models with different groups of caregivers. It would also be valuable to examine whether associations persist when examined longitudinally. Changes might be expected, for example, where social support^42^ or closeness in the relationship^43^ decline, neuropsychiatric symptoms increase in number and severity, or the person with dementia moves into institutional care.^6^ Further waves of follow-up in IDEAL will provide this longitudinal perspective.

The model presented here indicates key predictors of caregivers’ capability to “live well”, comprising evaluations of QoL, satisfaction with life and well-being. This builds on previous research to provide a template for conceptualizing the elements that should be included when considering how best to support caregivers of people with mild-to-moderate dementia living in the community. Optimizing mental and physical health is vital. Beyond this, understanding the profile of psychological characteristics and how this influences each caregiver’s experience would make it possible to target support more precisely to those caregivers who would most benefit from it. This would include encouraging the development of effective coping strategies. Similarly, understanding the nature of each caregiver’s social networks and resources and how these change over time^42^ would highlight ways in which information and support could be augmented in order to meet important needs for connection with others^45^ and alleviate negative impacts of caregiving.

In conclusion, these findings present new evidence about the relative impact of different aspects of the experience of caregivers of community-dwelling individuals with mild to moderate dementia on caregivers’ QoL, satisfaction with life and well-being, incorporating a wider range of potential predictor variables than previously considered. The findings demonstrate the importance of supporting caregivers’ psychological and physical health and their ability to develop and maintain positive coping strategies, as well as enabling caregivers to maintain vital social capitals, assets and resources. Greater understanding of the contribution of these domains of experience to caregivers’ capability to “live well” will help to inform policy discussions and decisions about health and social care provision, so as to enhance the support available to caregivers of people with dementia.

## Supplementary Material

SUPPLEMENTARY MATERIAL

Supplemental Digital Content is available for this article. Direct URL citations appear in the printed text and are provided in the HTML and PDF versions of this article on the journal's website, www.alzheimerjournal.com.
