# Supplementary material for: A Comprehensive Model of Factors Associated With Capability to “Live Well” for Family Caregivers of People Living With Mild-to-Moderate Dementia: Findings From the IDEAL Study
Source: Alzheimer Dis Assoc Disord. 2018 Dec 5;33(1):29–35. doi: 10.1097/WAD.0000000000000285 (PMC6416095; doi:10.1097/WAD.0000000000000285)
Supplement: SUPPLEMENTARY MATERIAL [file wad-33-29-s001.docx]

**A comprehensive model of factors associated with quality of life, satisfaction with life and well-being for family carers of people living with mild-to-moderate dementia: findings from the IDEAL study**

**Supplementary information**

**Analytical methods**

The analysis was based on version 2.0 of the IDEAL dataset.

The analysis first investigated the relationships between individual measures and living well outcomes using linear regression modelling and adjusting for age, sex, dementia subtypes and type of caregiving relationship. This was used to quantify the strength and direction of individual associations. Within each of the seven domains reflecting carers’ perceptions of their personal resources and experiences (Supplementary Table 1), all variables were fitted in one multivariate regression model adjusting for age, sex, subtypes and type of carers. Based on the adjusted results, three selection criteria were applied to identify the variables most clearly related to life satisfaction (Satisfaction with Life Scale; SwLS), wellbeing (World Health Organization-Five Well-Being Index; WHO-5) and quality of life (World Health Organization Quality of Life-BREF; WHOQOL-BREF) and to simplify the model as much as possible:

(a) Statistical significance: Wald test was used to examine whether the associations between living well outcomes and a specific measurement achieved statistical significance.

(b) Meaningful difference: The effect sizes were considered to be meaningful when unstandardised regression coefficients achieved SwLS>1.5 or WHO-5>5.0. Since there is no cut-off for the WHOQOL-BREF factor score, this criterion only applied to the other two living well measures. These cut-offs were determined to address the need for clinical relevance and based on the literature.

(c) Binary/ordinal variables: If there was a dose-response relationship, the measure was used as an ordinal variable. Categorical variables were regrouped into binary variables if appropriate.

After the selection process using multivariate modelling, structural equation modelling (SEM) was employed to generate a latent factor for the selected variables within each domain and build a structural model examining the associations between individual latent factors and the living well latent with SwLS fixed at 1. The variances of individual latent factors were fixed at 1. Two domains had only one variable each: for Managing everyday life with dementia this was the Neuropsychiatric Inventory Questionnaire distress scale and for Relationship it was the Positive Affect Index assessing current relationship quality. The results of SEM for the other five domains are reported in Supplementary Table 2. A full model was fitted to include the five latent factors and two individual variables, and was adjusted for age, sex, dementia subtypes and type of caregiving relationship. To account for correlations between latent factors and stabilise estimates in the full model, loneliness was found to also be important in the experiencing caregiving domain. To enable the model to reflect a positive perspective on ‘living well’ the scales of the three living well measures were reversed. The results of the full model are reported in Supplementary Table 3. Multiple imputation was used to address missing data in selected variables and living well outcomes. The percentage of missing data was between 7% and 20% across all domains. Age, sex, dementia subtypes and type of caregiving relationship were also included in the imputation model. Since imputation of ten datasets is usually sufficient to address potential variability of coefficient estimates,^1^ ten imputed datasets were generated and combined using Rubin’s rule.^2^ All analyses were conducted using Stata 14.2.

Supplementary Table 1. Variables under each domain considered for inclusion in the structural equation model

| Variables | Scale/source and reference |
| --- | --- |
| SOCIAL CAPITALS, ASSETS AND RESOURCES | |
| Frequency of social contact | Office for National Statistics Social Capital Scale^3^ |
| Social network | Lubben Social Network Scale^4^ |
| Social resources | Resource Generator-UK^5^ |
| Social participation | Office for National Statistics Social Capital Scale^3^ |
| Civic participation | Office for National Statistics Social Capital Scale^3^ |
| Neighbourhood trust | Office for National Statistics Social Capital Scale^3^ |
| Neighbourhood willingness to help | Office for National Statistics Social Capital Scale^3^ |
| Education | Highest level of education achieved |
| Cultural activity | Questions from Cultural Capital and Social Exclusion Survey^6^ |
| SOCIAL LOCATION | |
| Socio-economic status | Socio-economic status based on occupation^7^ |
| Social comparison | Single item |
| Perceived status in society | MacArthur Scale of Subjective Social Status (social ladder)^8^ |
| Perceived status in community | MacArthur Scale of Subjective Social Status (community ladder)^8^ |
| PSYCHOLOGICAL CHARACTERISTICS AND HEALTH | |
| Personality | Mini-IPIP^9^ |
| Religion | Single item^10^ |
| Spirituality | Single item |
| Optimism | Life Orientation Test-Revised^11^ |
| Self-esteem | Rosenberg Self-Esteem Scale^12^; single item^13^ |
| Self-efficacy | Generalized Self-Efficacy Scale^14^ |
| Loneliness | De Jong Gierveld Loneliness Scale^15^; single item |
| Depression | Center for Epidemiologic Studies Depression Scale-Revised^16^ |
| Subjective age | Single item |
| Life events | Modified 10-item Social Readjustment Rating Scale^17^ |
| PHYSICAL FITNESS AND HEALTH | |
| Physical activity | General Practice Physical Activity Questionnaire^18^ |
| Falls | Number of falls in past year^19^ |
| Eyesight | Single item^19^ |
| Hearing | Single item^19^ |
| Alcohol consumption | Currently does/does not consume alcohol |
| Smoking | Current smoker/former smoker/never smoked |
| Self-rated health | Single item^20^ |
| Health conditions | Charlson Co-morbidity Index^21, 22^ |
| MANGING EVERYDAY LIFE WITH DEMENTIA | |
| Hours of care | Single item |
| Distress at neuropsychiatric symptoms | Neuropsychiatric Inventory Questionnaire^23^ |
| RELATIONSHIP WITH PERSON WITH DEMENTIA | |
| Current relationship quality | Positive Affect Index^24^ |
| Past relationship quality | Positive Affect Index^24^ |
| EXPERIENCING CAREGIVING | |
| Positive aspects of caregiving | Positive aspects of caregiving^25^ |
| Role captivity | Role captivity^26^ |
| Caregiving competence | Caregiving competence^27^ |
| Management of situation – firm | Management of situation^26^ |
| Management of situation – things | Management of situation^26^ |
| Management of situation – busy | Management of situation^26^ |
| Management of situation – learn | Management of situation^26^ |
| Management of meaning – experiences | Management of meaning^26^ |
| Management of meaning – comparisons | Management of meaning^26^ |
| Management of meaning – larger sense | Management of meaning^26^ |
| Social restriction | Modified Social Restriction Scale^28^ |
| Stress | Relative Stress Scale^29^ |
| Coping | Single item^30^ |

**Supplementary Table 2.** Results of structural equation modelling for the five latent factors

**(a) Social capitals, assets and resources (CAR)**

|  | Model 1 | Model 2 | |
| --- | --- | --- | --- |
| **Measurement: living well** |  |  | |
| SwLS | 1 | 1 | |
| WHO-5 | 3.32 (3.07, 3.57) | 3.32 (3.07, 3.58) | |
| WHOQOL-BREF | 0.41 (0.38, 0.44) | 0.39 (0.36, 0.42) | |
|  |  |  | |
| **Measurement: Capitals, assets and resources** | | |  |
| Frequency of social contact |  |  | |
| Ordinal variable | 0.53 (0.42 0.65) | 0.66 (0.56, 0.76) | |
| Civic participation |  |  | |
| High vs low (ref.) | 0.06 (0.02, 0.09) | 0.05 (0.01, 0.09) | |
| Social resources |  |  | |
| Continuous variable | -3.50 (-4.18, -2.81) | -2.84 (-3.34, -2.34) | |
|  |  |  | |
| **Structural** |  |  | |
| CAR -> Living well | 1.68 (1.24, 2.12) | 2.16 (1.65, 2.67) | |

Model 1: unadjusted; Model 2: adjusted for age, sex, subtypes and type of carers

**(b) Social location (SLC)**

|  | Model 1 | Model 2 | |
| --- | --- | --- | --- |
| **Measurement: living well** |  |  | |
| SwLS | 1 | 1 | |
| WHO-5 | 3.33 (3.09, 3.57) | 3.34 (3.10, 3.58) | |
| WHOQOL-BREF | 0.41 (0.38, 0.44) | 0.40 (0.37, 0.43) | |
|  |  |  | |
| **Measurement: Social locations** | | |  |
| Social comparison |  |  | |
| Ordinal variable | 0.40 (0.31, 0.48) | 0.43 (0.29, 0.57) | |
| Societal ladder |  |  | |
| Ordinal variable | 0.64 (0.58, 0.70) | 0.63 (0.54, 0.72) | |
| Community ladder |  |  | |
| Ordinal variable | 0.54 (0.49, 0.59) | 0.53 (0.46, 0.60) | |
|  |  |  | |
| **Structural** |  |  | |
| SLC -> Living well | 2.19 (1.71, 2.68) | 2.24 (1.55, 2.93) | |

Model 1: unadjusted; Model 2: adjusted for age, sex, subtypes and type of carers

**(c) Psychological characteristics & health (PSY)**

|  | Model 1 | Model 2 | |
| --- | --- | --- | --- |
| **Measurement: living well** |  |  | |
| SwLS | 1 | 1 | |
| WHO-5 | 3.32 (3.08, 3.56) | 3.33 (3.09, 3.57) | |
| WHOQOL-BREF | 0.39 (0.36, 0.42) | 0.39 (0.36, 0.42) | |
|  |  |  | |
| **Measurement: Psychological characteristics & health** | | |  |
| Personality- Neuroticism |  |  | |
| Continuous variable | 2.24 (2.06, 2.42) | 2.25 (2.08, 2.44) | |
| Optimism |  |  | |
| Continuous variable | -2.45 (-2.67, -2.22) | -2.43 (-2.66, -2.20) | |
| Depression |  |  | |
| Yes vs No (ref.) | 0.19 (0.17, 0.22) | 0.19 (0.17, 0.22) | |
| Subjective age |  |  | |
| Ordinal variable | -0.27 (-0.31, -0.23) | -0.27 (-0.31, -0.23) | |
| Self-esteem (Rosenberg) |  |  | |
| Ordinal variable | -0.73 (-0.78, -0.69) | -0.72 (-0.78, -0.68) | |
| Loneliness |  |  | |
| Ordinal variable | 0.37 (0.33, 0.40) | 0.36 (0.32, 0.40) | |
|  |  |  | |
| **Structural** |  |  | |
| PSY -> Living well | -4.41 (-4.75, -4.07) | -4.45 (-4.80, -4.10) | |

Model 1: unadjusted; Model 2: adjusted for age, sex, subtypes and type of carers

**(d) Physical fitness & health (PHY)**

|  | Model 1 | Model 2 | |
| --- | --- | --- | --- |
| **Measurement: living well** |  |  | |
| SwLS | 1 | 1 | |
| WHO-5 | 3.34 (3.11, 3.58) | 3.37 (3.13, 3.60) | |
| WHOQOL-BREF | 0.47 (0.43, 0.51) | 0.45 (0.41, 0.48) | |
|  |  |  | |
| **Measurement: Physical fitness & health** | | |  |
| Eyesight |  |  | |
| Ordinal variable | 0.38 (0.33, 0.44) | 0.39 (0.33, 0.44) | |
| Self-rated health |  |  | |
| Ordinal variable | 0.71 (0.64, 0.78) | 0.70 (0.63, 0.76) | |
| Smoking |  |  | |
| Ordinal variable | 0.09 (0.05, 0.13) | 0.09 (0.05, 0.13) | |
|  |  |  | |
| **Structural** |  |  | |
| PHY -> Living well | -3.07 (-3.45, -2.69) | -3.19 (-3.58, -2.81) | |

Model 1: unadjusted; Model 2: adjusted for age, sex, subtypes and type of carers

**(e) Experiencing caregiving (EC)**

|  | Model 1 | Model 2 | |
| --- | --- | --- | --- |
| **Measurement: living well** |  |  | |
| SwLS | 1 | 1 | |
| WHO-5 | 3.28 (3.03, 3.53) | 3.28 (3.03, 3.53) | |
| WHOQOL-BREF | 0.37 (0.35, 0.40) | 0.37 (0.34, 0.40) | |
|  |  |  | |
| **Measurement: Experiencing caregiving** | | |  |
| Stress |  |  | |
| Ordinal variable | 0.76 (0.72, 0.79) | 0.75 (0.71, 0.78) | |
| Role captivity |  |  | |
| Ordinal variable | 0.54 (0.50, 0.58) | 0.55 (0.51, 0.59) | |
| Social restriction |  |  | |
| Ordinal variable (ref.) | 0.27 (0.22, 0.32) | 0.28 (0.23, 0.33) | |
|  |  |  | |
| **Structural** |  |  | |
| EC -> Living well | -3.42 (-3.77, -3.06) | -3.39 (-3.75, -3.03) | |

Model 1: unadjusted; Model 2: adjusted for age, sex, subtypes and type of carers

**Supplementary Table 3.** Results of structural equation modelling including all five latent factors, neuropsychiatric inventory distress scale (NPI) and current relationship quality (CR)

|  | Unadjusted | Adjusted |
| --- | --- | --- |
| Measurement model (LW) |  |  |
| SwLS | 1 (fixed) | 1 (fixed) |
| WHO-5 | 3.43 (3.19, 3.66) | 3.42 (3.19, 3.66) |
| WHOQOL-BREF | 0.40 (0.38, 0.43) | 0.40 (0.38, 0.43) |
| Structural association |  |  |
| PSY | 2.54 (2.15, 2.93) | 2.53 (2.08, 2.97) |
| PHY | 1.37 (1.06, 1.68) | 1.48 (1.04, 1.91) |
| EC | 1.32 (0.97, 1.66) | 1.34 (0.99, 1.70) |
| CAR | 0.58 (0.34, 0.83) | 0.68 (0.35, 1.00) |
| SLC | 0.08 (-0.17, 0.34) | 0.28 (-0.33, 0.89) |
| NPI | 0.08 (-0.13, 0.28) | 0.06 (-0.15, 0.28) |
| CR | -0.21 (-0.40, -0.03) | -0.22 (-0.41, -0.03) |
| Correlation/Covariance |  |  |
| (PSY, PHY) | 0.54 (0.45, 0.62) | 0.54 (0.46, 0.62) |
| (PSY, EC) | 0.55 (0.50, 0.61) | 0.56 (0.50, 0.61) |
| (PSY, CAR) | 0.27 (0.18, 0.36) | 0.26 (0.17, 0.35) |
| (PSY, SLC) | -0.48 (-0.57, -0.38) | -0.59 (-0.84, -0.34) |
| (PSY, NPI) | 0.36 (0.29, 0.43) | 0.36 (0.29, 0.43) |
| (PSY, CR) | -0.36 (-0.43, -0.30) | -0.36 (-0.43, -0.30) |
| (PHY, EC) | 0.26 (0.17, 0.34) | 0.26 (0.18, 0.34) |
| (PHY, CAR) | 0.28 (0.18, 0.38) | 0.28 (0.18, 0.39) |
| (PHY, SLC) | -0.42 (-0.52, -0.31) | -0.53 (-0.77, -0.28) |
| (PHY, NPI) | 0.17 (0.09, 0.24) | 0.17 (0.09, 0.25) |
| (PHY, CR) | -0.10 (-0.19, -0.02) | -0.11 (-0.19, -0.02) |
| (EC, CAR) | 0.07 (-0.03, 0.18) | 0.05 (-0.05, 0.16) |
| (EC, SLC) | -0.20 (-0.30, -0.11) | -0.31 (-0.57, -0.05) |
| (EC, NPI) | 0.64 (0.58, 0.69) | 0.64 (0.58, 0.69) |
| (EC, CR) | -0.56 (-0.62, -0.50) | -0.56 (-0.62, -0.50) |
| (CAR, SLC) | -0.35 (-0.45, -0.26) | -0.33 (-0.46, -0.20) |
| (CAR, NPI) | -0.04 (-0.13, 0.05) | -0.05 (-0.13, 0.04) |
| (CAR, CR) | -0.02 (-0.10, 0.07) | 0.00 (-0.09, 0.08) |
| (SLC, NPI) | -0.15 (-0.23, -0.06) | -0.23 (-0.43, -0.03) |
| (SLC, CR) | 0.17 (0.09, 0.24) | 0.22 (0.09, 0.35) |
| (NPI, CR) | -0.40 (-0.46, -0.34) | -0.40 (-0.46, -0.34) |

Adjusted for age, sex, subtypes and type of carers

References

1. von Hippel PT. How many imputations do you need? A two-stage calculation using a quadratic rule. *Sociological Methods & Research*. 2018.

2. Rubin DB. Multiple imputation after 18+ years. *Journal of the American Statistical Association*. 1996;91:473-489.

3. Office for National Statistics. *Harmonised concepts and questions for social data sources, secondary standards. Social capital*. Titchfield, UK: Office for National Statistics; 2008.

4. Lubben J, Blozik E, Gillmann G, et al. Performance of an abbreviated version of the Lubben Social Network Scale among three European community-dwelling older adult populations. *Gerontologist*. 2006;46:503-513.

5. Webber MP, Huxley PJ. Measuring access to social capital: the validity and reliability of the Resource Generator-UK and its association with common mental disorder. *Soc Sci Med*. 2007;65:481-492.

6. Thomson K. *Cultural capital and social exclusion survey: technical report*. London: National Centre for Social Research; 2004.

7. Office for National Statistics. *Standard Occupational Classification 2010. Volume 3. The National Statistics Socio-economic Classification: (Rebased on the SOC2010) User Manual*. Basingstoke: Palgrave Macmillan; 2010.

8. Adler NE, Epel ES, Castellazzo G, et al. Relationship of subjective and objective social status with psychological and physiological functioning: preliminary data in healthy white women. *Health Psychol*. 2000;19:586-592.

9. Donnellan MB, Oswald FL, Baird BM, et al. The Mini-IPIP scales: tiny-yet-effective measures of the Big Five factors of personality. *Psychol Assess*. 2006;18:192-203.

10. Loewenthal KM, MacLeod AK, Cinnirella M. Are women more religious than men? Gender differences in religious activity among different religious groups in the UK. *Pers Individ Dif*. 2002;32:133-139.

11. Scheier MF, Carver CS, Bridges MW. Distinguishing optimism from neuroticism (and trait anxiety, self-mastery, and self-esteem): a reevaluation of the Life Orientation Test. *J Pers Soc Psychol*. 1994;67:1063-1078.

12. Rosenberg M. *Society and the adolescent self-image*. Princeton, NJ: Princeton University Press; 1965.

13. Robins RW, Hendin HM, Trzesniewski KH. Measuring global self-esteem: construct validation of a single-item measure and the Rosenberg self-esteem scale. *Pers Soc Psychol Bull*. 2001;27:151-161.

14. Schwarzer R, Jerusalem M. Generalized Self-Efficacy Scale. In: Weinman J, Wright S, Johnston M, editors. *Measures in health psychology: a user’s portfolio Causal and control beliefs*. Windsor, UK: NFER-NELSON; 1995. p. 35-37.

15. De Jong Gierveld J, Tilburg TV. A 6-item scale for overall, emotional, and social loneliness confirmatory tests on survey data. *Res Aging*. 2006;28:582-598.

16. Eaton WW, Smith C, Ybarra M, et al. Center for Epidemiologic Studies Depression Scale: review and revision (CESD and CESD-R). In: Maruish ME, editor. *The Use of Psychological Testing for Treatment Planning and Outcomes Assessment*. 3rd ed. Mahwah, NJ: Lawrence Erlbaum; 2004. p. 363-377.

17. Holmes TH, Rahe RH. The Social Readjustment Rating Scale. *J Psychosom Res*. 1967;11:213-218.

18. National Health Service. *The General Practice Physical Activity Questionnaire (GPPAQ): a screening tool to assess adult physical activity levels, within primary care*. London: Department of Health; 2009.

19. Marmot M, Oldfield Z, Clemens S, et al. English Longitudinal Study of Ageing: Wave 2 2004-2005. In: Service UD, editor. 27th ed2017.

20. Bowling A. Just one question: if one question works, why ask several? *J Epidemiol Community Health*. 2005;59:342-345.

21. Charlson ME, Charlson RE, Peterson JC, et al. The Charlson comorbidity index is adapted to predict costs of chronic disease in primary care patients. *J Clin Epidemiol*. 2008;61:1234-1240.

22. Charlson ME, Pompei P, Ales KL, et al. A new method of classifying prognostic comorbidity in longitudinal studies: development and validation. *J Chronic Dis*. 1987;40:373-383.

23. Kaufer DI, Cummings JL, Ketchel P, et al. Validation of the NPI-Q, a brief clinical form of the Neuropsychiatric Inventory. *J Neuropsychiatry Clin Neurosci*. 2000;12:233-239.

24. Bengtson VL, Schrader SS. Parent-child relations. In: Mangon DJ, Peterson WA, editors. *Research instruments in social gerontology: Social roles and social participation*. Minnesota: University of Minnesota Press; 1982. p. 115-185.

25. Tarlow BJ, Wisniewski SR, Belle SH, et al. Positive Aspects of Caregiving contributions of the REACH project to the development of new measures for Alzheimer’s caregiving. *Res Aging*. 2004;26:429-453.

26. Pearlin LI, Mullan JT, Semple SJ, et al. Caregiving and the stress process: an overview of concepts and their measures. *Gerontologist*. 1990;30:583-594.

27. Robertson SM, Zarit SH, Duncan LG, et al. Family caregivers’ patterns of positive and negative affect. *Fam Relat*. 2007;56:12-23.

28. Balducci C, Mnich E, McKee KJ, et al. Negative impact and positive value in caregiving: validation of the COPE index in a six-country sample of carers. *Gerontologist*. 2008;48:276-286.

29. Greene JG, Smith R, Gardiner M, et al. Measuring behavioural disturbance of elderly demented patients in the community and its effects on relatives: a factor analytic study. *Age Ageing*. 1982;11:121-126.

30. McKee KJ, Philp I, Lamura G, et al. The COPE index--a first stage assessment of negative impact, positive value and quality of support of caregiving in informal carers of older people. *Aging Ment Health*. 2003;7:39-52.
